# Editorial: AI and mobile technologies for population-level chronic disease prevention

**DOI:** 10.3389/fpubh.2026.1975680

**Published:** 2026-10-27

**Authors:** Han Feng, Yishi Jia, Yingshuo Liu, Xiang Li, Duo Yu

**Affiliations:** 1 Tulane Research Innovation for Arrhythmia Discovery (TRIAD), Tulane University School of Medicine, New Orleans, LA, United States; 2 Tulane Heart and Vascular Institute, Tulane University School of Medicine, New Orleans, LA, United States; 3 Division of endocrinology, diabetes, and metabolism, Department of Medicine, College of Medicine, University of Illinois Chicago, Chicago, IL, United States; 4 Division of Biostatistics, Data Science Institute, Medical College of Wisconsin, Milwaukee, WI, United States

**Keywords:** AI, chronic disease, continuous monitoring, digital health, disease prevention, wearable device

Although chronic diseases develop over years, prevention is still often organized around brief and widely spaced clinical encounters. Artificial intelligence (AI), smartphones, wearable sensors, telehealth, and digital platforms offer a different model, in which risk can be observed between visits, support can be delivered closer to everyday decisions, and services can be adjusted as needs change (1–6). The 23 contributions to this Research Topic examine this opportunity in the context of cardiovascular disease, diabetes, obesity-related risk factors, cognitive health, kidney disease, sleep disorders, rehabilitation, and chronic disease management. Together, these range from community studies and qualitative syntheses to prediction models, intervention evaluations, systematic reviews, and forward-looking discussions of health system implementation. The value of digital health does not simply lie in collecting more data or producing a more accurate algorithm. Rather, it lies in whether reliable information reaches the right person, leads to an appropriate and accessible action, and improves health without increasing burden or inequality. Viewed in this way, the contributions are organized into six connected themes (Figure 1): designing for real populations; extending measurement beyond the clinic; converting data into useful predictions; closing the loop from information to care; supporting behavior without creating digital harm; and building equitable, governed, and scalable systems.

**Figure 1:**
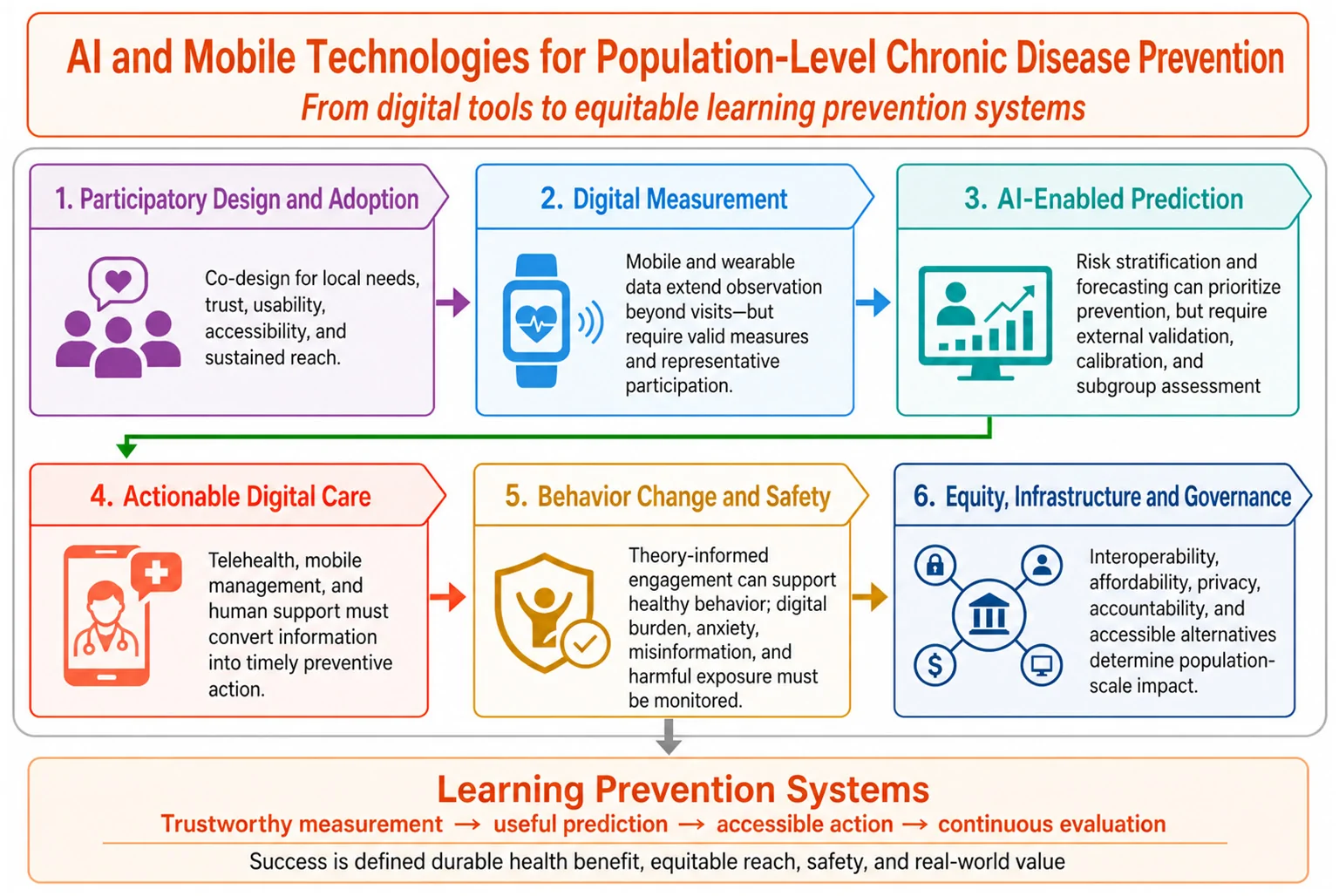
Central illustration of articles published under the theme “AI and Mobile Technologies for Population-Level Chronic Disease Prevention,” categorized into six themes: “Participatory Design and Adoption,” “Digital Measurement,” “AI-Enabled Prediction,” “Actionable Digital Care,” “Behavior Change and Safety,” and “Equity, Infrastructure, and Governance.”

## Designing for real populations

Population impact begins before an application is built. A lifestyle needs assessment in communities in Ireland and Romania (Iglesias-Cans et al.) showed that barriers, motivations, and preferred forms of digital coaching differ across local settings. This finding complements a survey-based model of digital health intention (Pu and Huang), in which expectations, health beliefs, and perceptions of persuasive design shaped willingness to adopt digital services. Both studies caution against equating access to a smartphone with readiness, trust, or sustained use.

The qualitative contributions deepen this point. A meta-synthesis of cardiovascular patients' experiences (Shao et al.) identified reassurance, human oversight, age-friendly design, and culturally responsive connections among families, hospitals, and communities as recurring needs. A protocol focused on young adults with cardiovascular disease (Cen et al.) took a life-course approach, asking how the possibilities offered by a technology are understood and used by a population with distinctive social roles and expectations. Although the latter has not yet reported its findings, it has highlighted a neglected issue: the same interface may enable independence for one group while creating surveillance, anxiety, or inconvenience for another.

These contributions place co-design, usability, and social context within the causal pathway of effectiveness. An intervention that is not reached, trusted, understood, or sustained cannot improve population health. Evaluations should therefore report not only outcomes among active users, but also who participates, who declines, who disengages, and why.

## Extending measurement beyond the clinic

Mobile and wearable technologies can make behavior and physiology visible in everyday life. In a community sample (Song et al.), wearable-derived sleep duration showed a non-linear association with hypertension, illustrating how objective longitudinal measures can refine risk characterization beyond coarse self-report. Similarly, the Terrapino application (Andel et al.) demonstrated the reach of mobile cognitive health assessment and identified modifiable factors associated with an Alzheimer's risk score among thousands of users.

These studies demonstrate the epidemiological potential of devices that individuals already carry or wear, but they also mark some important boundaries. Cross-sectional associations cannot establish that changing sleep reduces hypertension, and predicting a questionnaire-derived risk score is not the same as predicting incident dementia. Voluntary app users may also differ systematically from the wider population. Digital measurement is therefore most useful when it is validated against meaningful outcomes, is stable across devices, is generalizable to different demographic groups, and is connected to an intervention that can change the measured risk.

Two technically adjacent studies reinforce the importance of measurement quality. A pilot classification framework for voice disorders (Madill et al.) found that agreement was stronger for broad categories than for specific and perceptually ambiguous diagnoses. A YOLOv8 study (Guo et al.) achieved promising local performance in detecting and staging pressure injuries from clinical images. Although neither study directly evaluates population-level chronic disease prevention, both expose issues that apply throughout AI-enabled health research: algorithms inherit uncertainty in clinical labels, and impressive internal accuracy does not guarantee performance across patients, devices, or settings.

## Converting data into useful predictions

Several contributions explore AI as a means of stratifying risk or anticipating change. One electronic medical record model (Lin et al.) identified patients at risk of exercise intolerance after percutaneous coronary intervention, with strong internal discrimination and potentially useful negative predictive value. The Sugar Slay ecosystem (Rengarajan et al.) combined glucose forecasting, wearable data, gamification, personalized feedback, and caregiver support for type 1 diabetes. These systems illustrate complementary uses of prediction: prioritizing rehabilitation assessment at the service level and supporting near-term decisions in daily self-management.

However, prediction is an intermediate outcome, not a health benefit. A model may perform well in a local dataset but lose calibration when the prevalence, workflow, coding, or patient mix changes. A forecast may be accurate but arrive too late, demand an action that is unavailable, or increase cognitive burden. Before deployment, prediction systems need temporal and geographic external validation, subgroup assessment, prospective monitoring, and comparison with simpler rules or existing care. More importantly, studies must evaluate the complete chain from prediction to response, including whether an alert was seen, whether recommended actions were appropriate and completed, and whether outcomes improved.

The broader reviews in this Research Topic place these examples in context. Reviews of AI-supported chronic disease self-management (Du et al.) and AI in type 2 diabetes (Yu) described functions spanning monitoring, risk prediction, conversational support, multi-omics, continuous glucose monitoring, and personalized treatment. Their scope shows how quickly the field is expanding, while also revealing that technical development is advancing faster than evidence on long-term clinical effectiveness, affordability, and equitable implementation.

## Closing the loop from information to care

The strongest practical message across the Research Topic is that digital information produces value when it changes care. In type 2 diabetes, an AI- and big-data-supported mobile program (Xin et al.) translated risk estimates into individualized diet, exercise, and glucose-monitoring plans and reported modest improvements in glycemia, self-care, and quality of life compared with conventional nurse-led management. Because the allocation was not randomized, these results require confirmation, but the intervention illustrates a closed loop in which data inform action, and subsequent data allow support to adapt.

Other interventions show that useful digital transformation does not always require complex AI. App-based follow-up after arteriovenous-fistula creation (Chen et al.) was associated with earlier cannulation and shorter dialysis-catheter use than telephone follow-up. In a randomized study (Wang et al.), WeChat- and cloud-supported nursing improved early anticoagulation monitoring, knowledge, adherence, and satisfaction among patients receiving warfarin, although complications and readmissions did not decrease over 6 months. These studies suggest that timeliness, continuity, education, and access to professional review may be more important than the novelty of the delivery channel.

A meta-analysis of digital interventions for obstructive sleep apnea supports this conclusion. Passive monitoring (Xia et al.) did not improve several objective outcomes overall, whereas more active, synchronous, or theory-informed approaches appeared more promising. The evidence base remains small, but the implication is important: measurement alone rarely changes health. Digital interventions must alter capability, opportunity, motivation, treatment, or coordination—and should identify which of these mechanisms actually produced the observed benefit.

## Supporting behavior without creating digital harm

Behavior change is central to prevention, but engagement should not be confused with health benefit. A review of persuasive system design (Zhang K. et al.) found that chronic disease applications often emphasize reminders, tailoring, and task support, while using social support and credibility features less consistently. A scoping review of digital nudges (Taniar et al.) organized approaches across lifestyle domains and identifies gaps in population diversity and long-term evaluation. Together, these reviews support the need for more explicit behavior-change theory and clearer reporting of active components.

This Research Topic also illustrates why maximizing digital exposure is an unsafe objective. A prospective cohort study of individuals with lumbar disc herniation (Tong et al.) linked algorithm-driven exposure to heterogeneous disease-related short videos with poorer psychological and rehabilitation outcomes through proposed mediating pathways. Causal interpretation remains limited by non-random exposure and possible residual confounding, but the study expands the safety agenda beyond privacy and model error. Repetitive, alarming, contradictory, or poorly contextualized content may increase anxiety and undermine recovery.

Conversational AI offers both promise and responsibility. A co-designed large-language-model prototype for HIV prevention in South Africa (Govathson et al.) was experienced as usable, private, and less stigmatizing. Although outside the scope of chronic non-communicable disease prevention, this demonstrates how conversational systems may facilitate sensitive disclosure and prepare more focused encounters. Acceptability, however, is not diagnostic accuracy or clinical benefit. These tools require evaluation of harmful outputs, communication of uncertainty, escalation procedures, human review, and assessment of downstream service uptake.

## Building equitable, governed, and scalable systems

Four broad reviews make clear that population impact depends on the surrounding system. Discussions of emerging information technologies, DeepSeek in Chinese primary care, and AI in acute and critical care described opportunities in screening, decision support, follow-up, workflow, and resource allocation, while repeatedly identifying fragmented data, weak interoperability, uneven capacity, privacy concerns, and uncertain accountability (Feng et al.; Zhang J. et al.; Gao et al.). A review of AI-enabled chronic disease self-management (Du et al.) reached a similar conclusion from the patient side: personalization is valuable only when data quality, clinician integration, and sustained support are adequate.

Equity cannot be assessed only after deployment. Digital prevention may exclude patients through device cost, connectivity problems, language barriers, disabilities, health literacy issues, or distrust. It may also perform differently because sensor error, missing data, labels, baseline risk, and access to recommended services vary across groups. Studies should therefore examine the full reach-to-outcome pathway: eligibility, invitation, access, enrollment, continued use, measurement quality, model performance, receipt of action, and clinical benefit. Low-bandwidth, multilingual, accessible, caregiver-supported, voice-based, and non-digital options are not peripheral accommodations; they are components of a resilient population strategy.

Governance must be equally concrete. Data minimization, purpose limitation, access control, auditability, retention rules, vendor oversight, and incident response are baseline requirements. Explanations should be matched to decisions and audiences: patients need understandable choices, clinicians need guidance on uncertainty and escalation, and health systems need version history and subgroup performance data. “Human in the loop” is meaningful only when responsibility, response time, and the authority to override are defined.

## Summary and future directions

The next phase of research should evaluate complete pathways rather than isolated devices or models. First, predictive performance should be followed by prospective external validation and impact evaluation. Trials and pragmatic studies should compare AI-enabled care with simpler risk rules and well-delivered usual care, so that the incremental contribution of AI can be distinguished from extra contact or better organization.

Second, outcomes should extend beyond short-term engagement and process measures. Depending on the intervention, studies should assess sustained behavioral changes, disease incidence or progression, functional status, quality of life, adverse effects, caregiver burden, costs, and service use. Follow-up periods must be long enough to detect fading effects and delayed harms. Reporting should include absolute reach, attrition, missingness, and subgroup results, not just average outcomes among those who completed a study.

Third, implementation should be studied as part of effectiveness. Researchers should document workflow fit, alert overload, staff time requirements, training needs, interoperability, maintenance requirements, and what happens when an algorithm or vendor changes. Adaptive learning is desirable, but only with version control, monitoring for drift, and clear rules for recalibration or withdrawal. Community and frontline partnerships should guide both intervention design and the interpretation of inequities.

Across the 23 contributions, the field's direction is encouraging, but the evidence remains uneven. We know that mobile and wearable technologies can extend observation beyond visits, that AI can produce clinically relevant risk estimates, and that digitally supported care can improve selected short-term outcomes (7, 8). We know less about durability, transportability, cost-effectiveness, hard clinical outcomes, and the fair distribution of benefits and burdens. The central challenge is therefore not to build more standalone tools. It is to create learning prevention systems in which trustworthy measurement leads to effective action, that action is accessible to diverse populations, outcomes are fed back into improvement, and governance keeps the system accountable. By that standard, innovation is not defined by the volume of data collected or the novelty of an algorithm, but by the durable and equitable prevention it enables.
